# Metropolitan Extension

**Published:** 1899-10-21

**Authors:** 


### The Hospital

A Journal of

The Medical Sciences and Hospital Administration.

Vol. XXVII.—No. 682. Edited by Sir Henry Burdett, K.C.B., and Solomon C. Smith, M.D., M.R.C.P. [October 21, 1899.

### METROPOLITAN EXTENSION.

The London County Council is a body not overburdened with friends, and those it possesses are not entirely free from a tendency to be candid rather than polite. It follows, almost of necessity, that the efforts now being made by the Council to facilitate the daily journeys of workmen and workwomen to places remote from the great centres of employment are receiving a certain amount of adverse criticism; but we think it will be found on examination that these efforts, although possibly capable of being modified advantageously in respect of certain details, are nevertheless, on the whole, eminently worthy of public approval and support. The continued extension of the "wilderness of bricks and mortar" which constitutes material London has been a matter of admiration to some, and a source of complaint to others, from time immemorial; and both admiration and complaint are now being still more forcibly excited by the tendency of the contained population to overflow the boundaries of the "wilderness" itself. Feelings analogous to those now entertained and expressed by dwellers at Putney were entertained, if not expressed, six hundred years ago by the then suburban residents of "the shady village of Charing," and serious apprehension was felt, in the days of Queen Elizabeth, lest the growth of London should ultimately come to encroach upon the leafy solitudes of Islington. Fifty years since, Ravenscourt Park, near Hammersmith, was the almost rural residence of a wealthy private family; and Clapham Common was surrounded by stately mansions, of which the few that now remain will soon share the fate of the rest, and will give way to streets of semi-detached "villas." On every side of London the same thing may be witnessed; and yet all this growth of villadom hardly touches the fringe of a problem of enormous magnitude and of vital importance, the problem of "the housing of the working classes." London requires the services of a countless number of people whose wages do not enable them to aspire to villadom, who are still confined within a narrow area surrounding their work, and who, in this area, are constrained to live under conditions antagonistic alike to the laws of God and man, and equally prohibitory of health, of decency, and of cleanliness. The first attempts to grapple with the difficulty were by the erection of huge buildings, several storeys in height, which could be let in separate rooms, or in suites of rooms, to suitable tenants; and it was soon found that such buildings were easily filled, at rents which afforded a moderate return for the capital invested in them. But these rents were paid by persons of a better class than those for whom the buildings were intended, who, in their turn, simply went away to intensify overcrowding elsewhere. They were like the Irishman's heap of dirt, of which he proposed to rid himself by digging a hole and burying it. When the dirt was buried, the stuff dug out to make the hole remained, and continued to occupy the surface which the owner had intended to clear. Between the limitations of space, and the enormous value of the surface of London, it soon became manifest that successful housing on a large scale could only be accomplished outside of the metropolitan boundaries, and could only be rendered possible by a very considerable increase, and an equally considerable cheapening, of the means of locomotion. Much has been done in both these directions by the County Council since they obtained possession of the existing lines of tramways, on which the fares have been reduced, and night services established; but, even now, land is too costly within the county to afford facilities for the construction of wholesome and comfortable dwellings for working people, and hence the prices within the range of the tramways are prohibitory. The London County Council is believed to have opened negotiations with County Councils and other authorities beyond the metropolitan boundary, at which its own powers abruptly cease, and to have made arrangements by which, when its own tramways are carried to this boundary, they will be continued beyond it by the adjacent authorities, and taken to places in which comparatively cheap land may be obtained, and comparatively cheap cottages erected. It need not be matter for surprise if the proprietors of handsome suburban houses, with grounds abutting upon the roads which the new lines will traverse, exclaim loudly that their property will be injured and their comforts impaired. They must make up their minds to bow before the inexorable necessities of the case; and their exclamations will be of no more avail than was Mrs. Partington's mop against the Atlantic.

They must retire before the advancing wave, as the dwellers at Charing and at Islington have retired before them. Their property will be enhanced in money value by its application to new uses; and it is as easy for a prosperous man of business to live now at Weybridge or Chertsey, or a score of other places of like kind, as it was for his father or grandfather to live at Putney or at Wimbledon. *Salus populi suprema lex;* and we live in an age in which everything is possible—except the arrest of inevitable progress.

